# Should We Provide Life-Sustaining Treatments to Patients with Permanent Loss of Cognitive Capacities?

**DOI:** 10.5041/RMMJ.10081

**Published:** 2012-07-31

**Authors:** Ofra G. Golan, Esther-Lee Marcus

**Affiliations:** 1 The Gertner Institute for Epidemiology & Health Policy Research, Sheba Medical Center, Tel Hashomer, Israel; and; 2 Geriatric Division, Herzog Hospital affiliated to the Hadassah-Hebrew University Faculty of Medicine, Jerusalem, Israel

**Keywords:** Dementia, ethics, life-sustaining treatment, minimally conscious state, persistent vegetative state, solidarity

## Abstract

A very troubling issue for health care systems today is that of life-sustaining treatment for patients who have permanently lost their cognitive capacities. These include patients in persistent vegetative state (PVS), or minimally conscious state (MCS), as well as a growing population of patients at the very end stage of dementia. These patients are totally dependent on life-sustaining treatments and are, actually, kept alive “artificially.” This phenomenon raises doubts as to the ethics of sustaining the life of patients who have lost their consciousness and cognitive capacities, and whether there is a moral obligation to do so. The problem is that the main facts concerning the experiences and well-being of such patients and their wishes are unknown. Hence the framework of the four principles—beneficence, non-maleficence, autonomy, and justice—is not applicable in these cases; therefore we examined solidarity as another moral value to which we may resort in dealing with this dilemma.

This article shows that the source of the dilemma is the social attitudes towards loss of cognitive capacities, and the perception of this state as loss of personhood. Consequently, it is suggested that the principle of solidarity—which both sets an obligation to care for the worst-off, and can be used to identify obligations that appeal to an ethos of behavior—can serve as a guiding principle for resolving the dilemma. The value of solidarity can lead society to care for these patients and not deny them basic care and life-sustaining treatment when appropriate.

## INTRODUCTION

One of the very troubling issues for health care systems today is that of life-sustaining treatment for patients who have permanently lost their cognitive capacities (PLCC patients). In this paper the definition of “life-sustaining treatment” is “treatment without which the patient will most likely die within six months, while he would live substantially longer with the treatment.”^1^ PLCC patients include patients in a persistent vegetative state (PVS), or minimally conscious state (MCS) in cases where there is no estimated chance for recovery. Also included in this category is a growing population of patients at the very end stage of dementia (as will be defined later). These conditions should not be confused with the state of brain death, which is the irreversible cessation of all function of the entire brain, including the brain-stem. PLCC patients, besides their full dependency on nursing care, need artificial feeding; most would occasionally get infections requiring antibiotics or suffer from other conditions that require routine medical treatment, and some are also dependent on oxygen supply or medical equipment like ventilators to substitute for essential body functions. This phenomenon has vast social and ethical implications, raising the question of what is the morally right treatment for these patients. Due to its limited scope, this article will not deal with the question of to what extent society should allocate limited resources for the administration of costly treatments to sustain the lives of such patients.

Obviously, the reason for saving and sustaining people’s lives is embodied in the philosophy of the intrinsic value of human life. Yet, the reality of such patients, who seem to have lost the pre-eminence of man above a beast, being kept alive “artificially,” creates a great dissonance with the philosophical concept that life is life is life. These doubts lead us to resort to the principle of solidarity for guidance as to the care that should be provided for such patients. For the purposes of this paper, the discussion relates merely to the moral question of whether life-sustaining treatment should or should not be provided, regardless of who should pay for this treatment, which may vary between health systems.

Solidarity is a fundamental value that has various meanings and culture-relative interpretations, but one of its moral contents, most relevant to our discussion and strongly emphasized by personalism,^2^ is that it requires concern for the well-being of the worse-off members of the group.^3^,^4^ As such, society should apparently do anything feasible to provide the necessary health care for such disadvantaged populations of incompetent patients, who “are among the very neediest people on earth.”^5^

But is life-sustaining really indicated by the principle of solidarity in these situations? Is it the most appropriate care for these patients? Is this what they would desire if they were able to speak for themselves?

## WHAT IS PLCC AND WHY IS IT DIFFERENT FROM OTHER TERMINAL DISEASES?

### Conditions of Permanent Cognitive Incompetence

A few medical conditions may involve permanent loss of cognitive capacities:^6^,^7^

- Persistent vegetative state—Patients in PVS regain phenomenal sleep–wake cycles, but their motor, auditory, and visual functions are restricted to mere reflexes and are definitely non-functional.
- Minimally conscious state—Patients in MCS manifest fluctuating signs of purposeful behavior, may follow simple commands, show gestural or verbal yes/no responses regardless of accuracy, and/or may verbalize intelligibly.
  Depending on the cause and course, some patients with PVS or MCS may regain consciousness to a certain extent; the discussion in this paper is limited to those with no or a negligible chance of recovery.
- Severe dementia—End stage of mental deterioration, characterized by loss of cognitive capacity. At the final stage of Alzheimer’s disease, the condition involves loss of control of bodily functions and motor powers. The patients sink into a state of relative mutism and unresponsiveness, neglecting all external stimuli or inner needs, and their continuing existence depends entirely on nursing care.^8^

Patients in PVS show no sign of consciousness; this is less so for patients in MCS or with severe dementia. However, it is not certain that a patient is unaware and incapable of experiencing. Basically, “there is an irreducible biologic limitation to knowing the conscious life of another person.”^7^ Moreover, recent functional magnetic resonance imaging (fMRI) findings show that the clinical examination, at times, may be insensitive to the presence of awareness.^7^

### Reasons for Questioning the Appropriateness of Life-Sustaining Treatment for PLCC Patients

The issue of life-sustaining treatment for PLCC patients has been discussed in many forums and in various societies, both as a policy issue and in relation to specific patients (for review of United States and British case law see Standler^9^ and Rifkinson-Mann^10^). The source of doubt whether PLCC patients should be provided such treatment may be the fact that just a few decades ago such patients would have died either from their original brain damage^6^ or, in the case of end-stage dementia, from malnutrition and dehydration due to their inability to swallow. In these circumstances “concern had been growing that some technologies designed to save lives … appeared in some patients to do no more than *extend the dying process.*”^11^ However, unlike patients at the end stage of other terminal illnesses, PLCC patients do not look like they are in “the dying process”; neither do they, generally, seem to be suffering, and their life expectancy is often unpredictable. Furthermore, the use of the term “dying” in relation to PLCC patients calls for considerable attention, as noted during the discussion of the issue at the President’s Council on Bioethics:^12^

Is the state of affairs described as being suspended between life and death really distinguishable from a state of affairs described simply as being alive, but being in very bad shape as a result of, say, a severe dementia or some other conditions?

Hence, though some philosophers would define loss of higher brain function as death,^13^,^14^ it seems that our society’s struggle with the appropriateness of life-sustaining treatment for PLCC patients stems from the perception that living in such state is worse than death.

### Social Attitudes towards Cognitively Incompetent Patients

#### Loss of cognitive capacities is regarded as death or worse

The loss of cognitive capacities is a horrific condition. There is no agreement as to the evaluation of “how dead or how alive” are patients in such states.^15^ Moreover, some claim that even if they are alive, they do not “have a life.”^15^ In several studies over time and across cultures, PVS was perceived as worse than death even among physicians.^6^,^16^,^17^ Being in a chronic MCS was considered worse than PVS,^6^ and dementia was described as “a human condition that we wish to avoid above all others.”^5^ Yet, these extreme expressions should be examined very carefully before applying them as guidance for actual treatment decisions regarding those captured in this state. Special caution is required when decisions to withdraw life-sustaining treatment from PLCC patients, who had not made any specific statements as to their wishes in the event of vegetative survival, are “based on what most reasonable people would want in these circumstances.”^17^

Firstly, we should examine *what is meant by saying that such a state is worse than death.* It is quite common to take these words literally, namely, that PLCC patients should not be kept alive since they would rather be dead. However, this interpretation may not reflect precisely what people actually mean by this term. Neither does it necessarily reflect people’s attitudes towards life-sustaining interventions.^18^ A study which examined preferences for life-sustaining treatment in 341 participants from Seattle with diverse health states revealed that there was not complete concordance between the rating of certain health states as worse than death and rejection of treatment in that state.^19^ Indeed, in 71 instances, participants rated health states as worse than death *but wanted treatment*. Discussions about these discordances led to a change of preference almost two-thirds of the time once the relation between treatment preference and health state rating was made explicit. In 23% of the cases they changed their health state rating to make the two concordant.^19^ Thus, it may be suggested that the statement that this condition is “worse than death” should be understood as a perception with no practical consequences. Either way, it should be noted that many people do not share this view. In the aforementioned study, permanent coma was rated as worse than death by 52% of the participants, but *in the group of nursing home residents only 28% rated this state as worse than death*. In fact, 31% of all participants rated coma as better than death.

Much more alarming evidence on the gap between the perceptions of relatively healthy people and actual patients about such health states is to be found in studies of locked-in syndrome (LIS) patients. These patients are aware and awake but cannot move or communicate verbally due to complete paralysis of nearly all voluntary muscles in the body (usually except the eyes). Contrary to the views of healthy individuals and medical professionals that such patients’ quality of life is so poor that it is not worth living, LIS patients typically self-report meaningful quality of life, and their demand for euthanasia is surprisingly infrequent.^20^

#### For whom is PLCC worse than death?

Another question is: for whom is PLCC worse than death? In the European survey, for example, participants agreed that valuing a vegetative state (VS) as worse than death was more relevant for the patient’s family than for the patient him/herself. This shows that PLCC is perceived as much more burdensome for those surrounding the patients and for society at large than it is for the patients themselves. So in examining the claim that PLCC patients should not be tortured by being kept alive with no hope of recovery, one should be very careful “to think whether we’re quite certain it’s the patient who’s being tortured or us.”^12^ It is important to acknowledge that we may sometimes have a problem with such patients’ presence; in Professor Meilaender’s words in relation to patients with advanced dementia, “there’s a part of us, there’s a part of me that inevitably wishes they’d go away not because it’s such a problem, but because they’re one of us. They show us our future, and they make us very uneasy.”^12^

#### Social attitudes towards loss of cognitive capacities and the perception of personhood

Stephen Post suggests that “we live in a culture that is … dominated by heightened expectations of rationalism and economic productivity, so clarity of mind and productivity inevitably influence our sense of the worth of a human life.”^5^ In such “hypercognitive culture”^5^ it is only natural that loss of cognitive capacities may be perceived as loss of personhood.

Different approaches to personhood have implications for the definition of PLCC patients as “persons” or “non-persons.” For those who advocate that it is necessary to possess certain cognitive capacities to qualify as a person, PLCC patients would not be regarded as such. Yet, they are definitely persons within the perception of inherent/ transcendental personhood, for which being a human is equated with being a person. According to interpersonal theories, their personhood depends on its recognition by others.^21^

The recognition of PLCC patients as persons is relevant to the question whether these patients should be treated like their fellow dependent cognitively competent patients, or differently; namely, whether they should or should not be offered life-sustaining treatment when such treatment would be offered to other dependent patients.

## IS THERE A MORAL OBLIGATION TO PROVIDE LIFE-SUSTAINING TREATMENT TO PLCC PATIENTS?

### Good Ethics Starts with Good Facts

The preliminary guiding principle of any ethical deliberation is that good ethics starts with good facts. In this discussion, however, there are more mysteries than facts.

*We know* that PLCC patients are human beings, that some are sentient, and that their life depends on on-going medical care. We also know that most people would not wish to be kept alive in this state, which is regarded by our society as “worse than death”; and there are even cases in which we have the patient’s advance directives not to be kept alive in such circumstances. Yet, *we do not know for certain* that they lack consciousness^22^ or fail to perceive pain.^10^,^23^ Moreover, *we do not have a clue* as to the experiences of PLCC patients: Do they have any feelings? Are they aware of their situation? Are they miserable or content? Do they feel deprived of their lost abilities or humiliated by such losses? Do they have any wishes? Do they wish to be kept alive, or to die? What are their spiritual experiences? Is their soul free or locked in? What if the loss of self-awareness spares these people from the fear of mortality towards the end their life? What if it is a release to the present from anxiety over the past and future, as a Japanese writer (Ariyoshi) has described advanced dementia?^5^ What if this situation releases them to a higher spiritual level? Maybe as high as one of the definitions of Zen: “being free of the distractions and illusory conflicts of the material world”?^24^ Do the alarming fMRI findings in a VS patient “indicate the existence of a rich mental life, including auditory language processing and the ability to perform mental imagery tasks,” as Naccache suggests in his *Science* commentary?^25^ Or is this “dramatic assertion” “simply unjustifiable,” as Fins and Schiff claim?^26^

These uncertainties create an epistemic gap. Thus, any decision to let a PLCC patient die must be taken very cautiously, especially due to the weight people attribute to even the slightest cognitive awareness; several studies showed that medical caregivers paradoxically supported treatment withdrawal much less in MCS compared to VS, although most of them recognized MCS as worse than VS and would not wish to be kept alive in this state.^6^,^27^,^28^

We should also be very careful not to interpret the experience of people with PLCC according to *our* values and ideals. Post notes that “people who become old and frail and unable to do all they could in the past tend to find unanticipated value in small gratifications.”^5^ As a result, he warns us in relation to demented patients,

we must not interpret the experience of people with dementia against a background ideal of pure reason and self-control ... If the pictures are sketched with achievement-oriented, socioeconomic, and cognitive values in mind, harm will result.^5^

Of course, there may be a great difference in this regard between patients with severe dementia, whose cognitive capacities deteriorated step by step, and other patients who suffered a sudden loss of their cognition, where there may be slight hope for a “miracle recovery,” depending on the cause of the event. Nevertheless, both states need to be examined with great humbleness, acknowledging our ignorance and inability to appreciate the subjective experience of PLCC patients, so that it may be wrong to apply to them our perceptions of this situation for better or worse.

### Analyzing the Dilemma

In dealing with the issue at stake, it would be advisable to use Pellegrino’s moral algorithm for ethical decisions to withdraw life-sustaining treatment.^29^ This algorithm contains three levels: (1) at the most fundamental level are the presuppositions about the value of human life; (2) at the intermediate level, a theoretical structure for justifying ethical decisions; and (3) at the practical level, a framework for actualizing the first two levels in a concrete decision. According to the algorithm, the main practical ethical questions that must be answered in any clinical decision to withhold or withdraw life-sustaining treatment are “Who decides?” and “By what criteria?” Our discussion will concentrate on the latter.

### The Value of Human Life

The value of human life “may be interpreted as absolute, relative, or instrumental.”^29^ If taken as an absolute value, life must be sustained at all costs, while at the other extreme, the lives of PLCC patients can be perceived as lacking instrumental value, and therefore they may be left to die. Under the relative interpretation “human life has enormous intrinsic value; therefore, we cannot dispose of it at our will when it loses instrumental value. But in view of our inevitable human finitude, under certain specific conditions”^29^ there may be no moral obligetion to provide life-sustaining treatment. Usually such specific conditions would be recognized when there is a disproportionate relationship between the burdens and the effectiveness or benefits of treatment. However, the case of PLCC patients might be different, since the views about sustaining their lives stem to a great extent from how people see them^15^ (see also the relationship to patients with dementia in the study of Skog et al.^30^). For those who consider PLCC patients as non-persons, loss of cognitive capacities per se might be regarded as specific circumstances in which life has a lesser value. This is the case for the unacceptable view of life unworthy of being alive (*lebensunwertes Leben*), as well as for other, less offensive philosophical views for which “what does have intrinsic value … is not biological life in itself, but the life of a human being in possession of at least a modicum of self-awareness and intellectual and other mental functioning.”^12^ Such life may be renounced, in line with, for example, John Harris’s argument that a person is a being capable of valuing its own existence, so taking the life of a non-person is not wrong, since it does not deprive them of anything they value.^31^ Yet, for those who see an intrinsic, though not absolute, value in the life of every human being, further investigation is necessary in order to determine if there are any conditions under which we should not or may not preserve life of PLCC patients.

### Decision Framework at the Practical Level

Generally, the application of life-sustaining treatments may pose a dilemma when there are grounds to assume that the burdens of the treatment for the patient might outweigh its benefits. This can happen either when the intervention itself is burdensome, or when the patient appreciates his/ her life to be so miserable that death is preferable. If the patient is competent, the decision would be made in respect of his/her autonomy.

Things tend to be much more complicated when the patient is incompetent to express his/her wishes. “When the patient lacks decision-making capacity, moral authority is transferred to a valid surrogate, a living will, or a durable power of attorney.”^29^ In such circumstances, decisions can be made according to the patient’s presumed will as far as this can be determined, based on his/her prospectively stated preferences, if there were any. When the patient’s subjective views are unknown, some jurisdictions apply the “best interests” standard, which adopts “the perspective of a ‘reasonable person’, choosing as most people would choose for themselves.”^7^ Other jurisdictions apply the presumption that a person wishes to continue living, unless proven otherwise (e.g. in Israel the dying patient law^32^) or the ethical rule, *in dubio pro vita*—“when in doubt, favor life.”^33^

### The Relevant Ethical Criteria

Two central conclusions can be drawn from the above outline: (1) that the core question is how we value the life of cognitively incapacitated patients; and (2) that the framework of the four principles—beneficence, non-maleficence, autonomy, and justice—may be applicable and helpful when the burdens and benefits of the treatment and the patient’s autonomous wishes are known or can be relatively accurately presumed. However, these ethical criteria are not straightforward in chronic disorders of consciousness due to the nature of the disorder.^1^ Therefore, there is a need to examine other moral values to which we may resort in dealing with this dilemma. Certain values, like care and the dignity of the human person, were suggested for the analysis of similar dilemmas.^34^ We suggest that the principle of solidarity, which is one of the values in European bioethics,^35^ could be used to promote the discussion and may offer some guidance.

## SOLIDARITY AS A GUIDING PRINCIPLE FOR RESOLVING THE DILEMMA

### The Concept of Solidarity

The term solidarity has been defined and employed in various ways by bioethicists or other academics working on bioethical questions over the last two decades.^36^ As per the working definition suggested in a report commissioned by the Nuffield Council on Bioethics, solidarity signifies “shared practices reflecting a collective commitment to carry ‘costs’ (financial, social, emotional, or otherwise) to assist others.”^36^ The definition consists of three tiers starting with a conceptualization of how individuals come to engage in practicing solidarity. At this level,

solidarity comprises manifestations of the willingness to carry costs to assist others with whom a person recognizes sameness or similarity in at least one relevant respect … It entails the awareness of being associated—by choice, by fate, or other circumstances, with others. It is, … an instance of seeing one’s own potential or actual fate, or that of loved ones, in the fate of another.^36^

Accordingly, in the current context, solidarity involves recognition of the respect(s) in which PLCC patients are similar to “us.” Such respects may be their being human creatures or their fate against which none of us is immune. A similar idea can be found in the Jewish thought as represented by the interpretation held by many commentators to the commandment “love thy neighbor as thyself”—“love thy neighbor (for) he is like yourself.”

The principle of solidarity is enacted especially with the most vulnerable in society, and in particular with those who cannot help themselves.^36^ Obviously PLCC patients are totally dependent on other people to care for all their needs; but, on the other hand, by definition they are (or are presumed to be) unaware of their situation, nor do they experience their weakness and dependence. Paradoxically, patients who have no cognitive capacities at all may be less vulnerable than others, less incapacitated. For example, the elderly demented are considered as “a weak group who cannot always administer their personal freedom on a par with others”^37^; but PLCC patients are most likely unable to form any autonomous will, thus they cannot feel deprived of the ability to administer their personal freedom. What follows is that we cannot tell if they would prefer to die or to be kept alive in their state; if life has any benefit for them, or if they would be better off dead. These circumstances cause doubts as to what course of action should be taken to answer the needs of these patients.

Caring for the worst-off is necessary *for us as a society*. “We care for the neediest because need is a basis of moral duty; the public weal is grounded in this moral sentiment.”^5^ This moral sentiment is known in European ethics as the fundamental value of solidarity;^4^ in American bioethics it may be referred to as the principle of equality,

the commitment to equal human worth stands as the basis of a welcoming community—one that assures all living human beings, even those in a disabled or diminished state, that their lives still have meaning, worth, and value for all of us. It assures them that we would not prefer them dead even if we would like to see an end to the suffering that marks their present condition.^8^

Apparently, the principle of equality requires society to respect the full dignity of PLCC patients. However, this may not be true for those who believe that “… for the human to be treated as a member in full standing of the human moral community—there must be integrated functioning of mind and body.”^13^

At any rate, the principle of equality “still leaves us with the difficult discernment of deciding what is truly in the best interests of patients,” as written by Erik Cohen in relation to persons with dementia.^38^ But, since we do not know most of the facts that are necessary to overcome this difficulty, we will try to seek guidance in further aspects of solidarity.

As indicated by Sass, solidarity “can also be used to identify interpersonal and professional duties and obligations that appeal to an ethos of behavior within that private or professional setting.”^39^ As such, solidarity may well lead us to focus on our care for PLCC patients, especially the elderly with end-stage dementia, as a reflection of the ethos of our society. Do we wish to live in a society that cares only for those who are capable of communicating and expressing their needs, or in one that cares for all its members all through their life cycle? Do we prefer an ethos of caring for those who are not even aware of how weak and helpless they are as much as they would have been cared for had they been conscious, or an ethos of withdrawing sustenance or life-maintaining care or of caring for such people as if they were already dead?

As articulated by (former) Vice-President of the Israeli Supreme Court, Menachem Elon, in the leading case in the issue under discussion,^40^ the ethos of Judaism is based on the concept of man’s creation in God’s image.^41^

The Torah begins with this, and Jewish law deduces from it fundamental principles about human worth—of every man as such—his equality and love, … we do not have the authority, nor do we have the right, to distinguish in any way whatsoever with regard to human worth between rich and poor, healthy and disabled, sane and insane. All human beings, because they were created in G-d’s image, are equal in their worth and quality.^40^

This principle has been accepted and is also used as a basis for the supreme value of human life in many different cultures and legal systems. It should be noted that this principle is not identical to the paradigm of vitalism according to which life should be maintained always, at any expense. Moreover, as Kasher says,

we have a “preciousness” conception of human life that does not rest on any view of the intrinsic value of human life or of a divinely endowed value of human life. This conception rests on the simple observation that being alive is a precondition for being a participant in societal arrangements that embody values and norms and distribute rights and duties. … According to this conception, protection of human life is protection of what is a necessary element of any valuable societal arrangement.^42^

In other words, any society which values solidarity should protect the life of all its members. Even if PLCC patients will not be deemed as “persons” in the full sense of the word, their moral status is very similar to a person, and we do have at least secondary moral duties towards them, since we encounter them “at a very high point on the slope of dignity protection.”^42^

### Applying the Concept of Solidarity to the Medical Care of PLCC Patients

The direct conclusion from the analysis above is that solidarity entails a moral obligation to give PLCC patients optimal medical and nursing care, using the same medical judgment and considerations as for any other dependent patient. This involves two perplexing issues—the suffering and dignity of the patient—which must be addressed in order to determine whether and which life-sustaining treatment is indeed the “best care” for a given patient (according to Cohen, “best care” encompasses both the solidarity and obligations of the caregiver to the patients, and the “best interests” of the patient).^8^,^38^ Life-sustaining treatments range from antibiotics and artificial nutrition and hydration to mechanical ventilation and dialysis. Each intervention should be considered separately according to the patient’s condition and prognosis, applying relevant evidence-based medicine. This means that the duty to sustain the lives of PLCC patients does not necessarily entail an obligation to use every available modality in every case.

If we accept as a guiding principle that the fact that the patient is mentally deficient does not make his/her life less worthy, these considerations should be taken into account just as they are considered for cognitively competent patients. Thus, any suffering entailed in the treatment and its outcomes should be given due weight. Certainly, if the patient is enduring pain and suffering that cannot be alleviated, it may be permitted and in certain circumstances even obligatory to refrain from prolonging life. However, in the case of PLCC patients, there is no indication that being in this state *as such* involves suffering; however notwithstanding, when a PLCC patient seems to be in pain or to be suffering otherwise, this should be adequately treated.^23^

Looking further into what might positively serve the well-being of the patient, it would be advisable to use the formula suggested by Jox,^1^ according to which, life-sustaining treatment should be continued if the well-being associated with this option is superior to the well-being associated with allowing the patient to die. Due to the epistemic gap regarding the well-being of PLCC patients, just as the value of (their) life after death, to which Jox relates in his formula, and since the only known parameter in the formula is that life in itself has a positive ethical value, it turns out that life-sustaining should be presumed to serve better the well-being of these patients. Moreover, compassionate care for such unresponsive patients is an expression of unconditional love, which is a great privilege for the caregivers, which might also give the patients an opportunity to experience (if and as much as they can) the feeling of this rare kind of love.

### The Dignity of the PLCC Patient

The dignity of the PLCC patient is a tougher issue, due to both the calls for “death with dignity” and the high value placed by Western society on cognition as an integral aspect of an individual’s dignity (in accordance with the Kantian reading, which sees dignity as based on rationality).^35^,^36^ However, dignity has other interpretations, relating to all human lives being created in the image of God, and having a human genome.^35^,^36^ In this regard it would be worthwhile to refer to two authorities from two different cultures that follow the principle of the equal worth of all human lives:

1. Nordenfelt described four notions of human dignity: the dignity of merit, the dignity of moral or existential stature, the dignity of identity, and the universal human dignity (*Menschenwürde*).^43^ The basic notion of dignity, *Menschenwürde*, which is the grounds for all human rights, pertains to all human beings to the same extent and cannot be lost as long as the person exists. By definition, the dignity of PLCC patients in this sense is definitely preserved. “According to this interpretation, loss of dignity cannot be used as an argument for euthanasia in persons with severe dementia.”^34^ Moreover, it may be plausible to argue that the same is true for loss of dignity according to the other interpretations just as well. Loss of dignity of merit is a common phenomenon, and loss of dignity of moral stature also happens sometimes, but by no means can they be used as an argument for euthanasia, not even in its passive form. Both kinds of dignity can come and go, but they can, on the other hand, continue to exist to some extent despite loss of cognitive capacities, at least as they do for the dead.
  Loss of dignity by PLCC patients relates to “dignity of identity” which “is tied to the integrity of the subject’s body and mind, and in many instances, although not always, also dependent on the subject’s self-image.”^34^ Yet, under this definition there is no difference between PLCC and other disabled patients! The latter’s loss of dignity may be even harder due to their preserved self-awareness. Hence they should be treated similarly.
2. Menachem Elon, of the Israeli Supreme Court, in citing the words of Ramsey, “the phrase dying with dignity is a contradiction in terms,” stressed that “There is a conflict between *the death* of a person and the *dignity* of a person. By contrast, the life of a human being is itself the dignity of man, and there is no conflict between the *life* and *dignity* of man, nor could there be a conflict.”^40^ Also, “Protection of human life is one dimension of protection of human dignity.”^42^

## CONCLUSIONS

As long as we know nothing about the subjective experience of PLCC patients, we may feel torn as to whether it means the person is more ready to die or whether it implies a special obligation to care.^38^ However, we all sympathize with the old prayer “Do not cast me off in the time of old age; forsake me not when my strength is spent”,^44^ a prayer that highlights the need for solidarity with the dependent members of society. The value of solidarity can lead any society that adheres to it to care for PLCC patients and not deny them basic care and life-sustaining treatment when appropriate. In light of Kasher’s analysis regarding neonates at the edge of viability, PLCC patients should be medically treated, in an ordinary way, unless there are compelling reasons for not treating them in an ordinary way or even at all (e.g. explicit advance directives). As the President’s Council for Bioethics stated,

we should remember that aiming at a person’s death is always a kind of betrayal; standing with the suffering person, in the hardest times, is not—even if we might rage together with the patient at the God, or nature, or universe that permits such misery, and even if we pray with the patient for an end to a painful life that is nevertheless not ours to end.^8^

Thus, with no indication that life in a state of PLCC is significantly burdensome for the patient, what we owe these patients—let alone patients in less extreme states of cognitive deficiency—is the same level of care, respectful for them and for their life, just as for any other person. The choice of which, and to what extent, life-sustaining treatment should be applied should be based on medical and ethical considerations in accordance with a compassionate approach to these patients. In specific cases, conflicting values and interests, like the burden for the family or for society at large, should receive due consideration resembling other similar dilemmas.

## Abbreviations:

fMRI: functional magnetic resonance imaging;
LIS: locked-in syndrome;
MCS: minimally conscious state;
PLCC: permanent loss of cognitive capacities;
PVS: persistent vegetative state;
VS: vegetative state.
